# Functional Immune Reconstitution by Interleukin-2 Adjunctive Therapy for HIV/Mycobacterial Co-infection

**DOI:** 10.3201/eid2109.150461

**Published:** 2015-09

**Authors:** Jan Heyckendorf, Sven Philip Aries, Ulf Greinert, Elvira Richter, Holger Schultz, Christoph Lange

**Affiliations:** Research Center, Borstel, Germany (J. Heyckendorf, U. Greinert, E. Richter, H. Schultz, C. Lange);; German Center for Infection Research, Borstel (J. Heyckendorf, U. Greinert, C. Lange);; Elbpneumologie, Hamburg, Germany (S.P. Aries);; Karolinska Institute, Stockholm, Sweden (C. Lange);; University of Namibia School of Medicine, Windhoek, Namibia (C. Lange)

**Keywords:** HIV, IL-2, immunotherapy, *Mycobacterium tilburgii*

**To the Editor:** We describe a case of an immunocompromised patient with AIDS who sought treatment for immunotolerance to an invasive, systemic mycobacterial infection that was unresponsive to antimycobacterial therapy alone. The 41-year old man sought treatment in November 2006 for fatigue, dyspnea, and epigastric pain of 4 weeks’ duration and weight loss of 10 kg. HIV-1 infection (20 cells/μL CD4+ T-cells, viral load 230,000 genome equivalents/mL) was diagnosed. Antiretroviral therapy (ART) and *Pneumocystis* pneumonia prophylaxis were initiated.

In June 2007, acid-fast bacilli (AFB) were detected on mediastinal lymph node specimens obtained by endobronchial-ultrasound-guided biopsy during a bronchoscopy; empiric antituberculosis treatment was initiated. *Mycobacterium tuberculosis* DNA was not detected by nucleic acid amplification on these specimens. At the time of referral to our clinic, the physical examination revealed generalized lymphadenopathy and oral leukoplakia. The patient’s bodyweight was 63 kg. Computed tomography showed extensive mediastinal and abdominal lymphadenopathy without other abnormalities. Serologic investigations showed negative results for hepatitis A, B, C, and syphilis. Esophageal-gastro duodenoscopy showed a cottage cheese–like appearance of the duodenal mucosa, and histopathological examination of biopsies documented massive numbers of AFB (Technical Appendix Figure, panel A). Nucleic acid amplification of 16S-rRNA from biopsies was performed, and sequence comparison to the National Center for Biotechnology Information database identified the presence of *M. tilburgii*. In July 2007, specific treatment against infection with *M.* *tilburgii* was initiated with rifabutin, ethambutol, and azithromycin (*1*).

Despite nondetectable levels of viral replication while the patient was receiving ART, CD4+ T cell count did not rise above 73 cells/μL (Figure). In November 2007, he reported diarrhea and weight loss of 6 kg (total weight 57 kg); testing showed hypochromic-microcytic anemia (hemoglobin 8.2 g/dL). Bone marrow biopsy showed infiltration of AFB, and 16S-rRNA amplification confirmed *M. tilburgii* infection. Macroscopic and microscopic appearance of the duodenal mucosa was unchanged.

**Figure:**
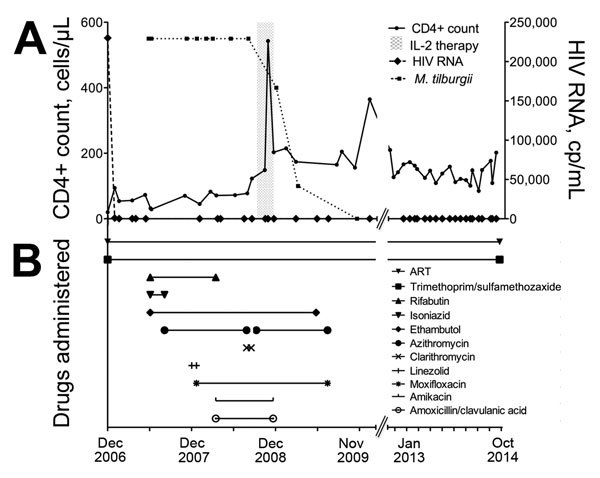
Laboratory findings and drug treatment regimen over time for an HIV-infected patient with disseminated *Mycobacterium tilburgii* infection, December 2006–October 2014. A) CD4+ T cell count, HIV viral load, and use of interleukin-2 (IL-2; gray shading). B) antimycobacterial drug combinations, antiretroviral therapy (ART), and trimethoprim/sulfamethoxazole prophylaxis.

During the next 10 months, antimycobacterial therapy had to be altered as a consequence of adverse drug events (Figure). In November 2007, treatment with linezolid resulted in an allergic reaction with generalized rash and fever. In March 2008, treatment with rifabutin was discontinued after pancytopenia developed. Treatment with amikacin between March and November 2008 resulted in hearing loss. During this time, the patient’s symptoms improved, and he gained 16 kg (total weight: 73 kg) when he received pulsed doses of prednisolone (20 mg/dL), but he had diarrhea when steroids were tapered to 10 mg/dL. By August 2008, after >1 year of antimycobacterial therapy, there were no improvements of clinical findings.

Adjunctive treatment with interleukin-2 (IL-2 [Proleukin S, Novartis Pharma GmbH, Nuremberg, Germany]) was administered subcutaneously (4.5 × 10^6^ IU) on 3 occasions in September, October, and November 2008. The mean post–IL-2 treatment CD4+ cell count was 242/μL, an improvement over 64/μL before the intervention (Figure). In November 2009, the duodenal mucosa appeared normal on inspection, and no bacteria were found on histopathological examinations (Technical Appendix Figure, panel B). Antimycobacterial therapy (Figure) was discontinued, steroid administration was gradually reduced, and measured bodyweight stabilized (72–74 kg). At the last examination in December 2014, the patient remained free of signs and symptoms of recurrence of *M. tilburgii* infection.

*M. tilburgii* is an uncultivable nontuberculous mycobacterium related to *M. simiae* and *M. genavense* (*2*). Fewer than 10 clinically relevant cases of *M. tilburgii* infections have been described in the literature (*2*–*7*); most were intestinal infections in immunocompromised hosts (*3*). Successful treatment has been achieved with combination regimens of antimycobacterial drugs that are also effective against *M. avium* complex (*4*).

In 2 studies that evaluated the effect of adjunctive IL-2 therapy in addition to ART for previously treatment-naive patients with HIV infection, baseline median numbers of circulating CD4+ cells increased significantly, but expansion of CD4+ T cells was not associated with the reduction in the risk for opportunistic diseases or death (*8*). In contrast to these results, in a study of HIV-positive patients who had low circulating CD4+ T cell counts, the participants experienced fewer AIDS-defining events and fewer deaths occurred when they were treated with adjunctive IL-2 immunotherapy (*9*).

This case report provides lessons for the understanding of mycobacterial diseases. First, despite massive infiltration of duodenal mucosa, mesenterial lymph nodes, and bone marrow, the lack of inflammatory responses in this patient prevented tissue destruction. Second, in the absence of a sufficient immune response and an increase in the number of circulating CD4+ T cells, antimycobacterial therapy without adjunctive immunotherapy did not clear the systemic bacterial infection.

Host responses to pathogens are not always beneficial. Intense immune reactions experienced during episodes of sepsis or HIV immune reconstitution inflammatory syndrome are frequently associated with patient death. Alternately, in the absence of inflammatory responses to pathogens, the patient is unprotected, and even microbiota that are harmless to an immunocompetent person can adversely invade. In an optimal immune response setting, a balance between proinflammatory and anti-inflammatory factors in response to pathogens is maintained (*10*).

Technical Appendix Duodenal biopsy histology showing effectiveness of therapy for HIV/mycobacterial co-infection.
