# Supplementary material for: Functional Immune Reconstitution by Interleukin-2 Adjunctive Therapy for HIV/Mycobacterial Co-infection
Source: Emerg Infect Dis. 2015 Sep;21(9):1685–7. doi: 10.3201/eid2109.150461 (PMC4550166; doi:10.3201/eid2109.150461)
Supplement: Technical Appendix — Duodenal biopsy histology showing effectiveness of therapy for HIV/mycobacterial co-infection. [file 15-0461-Techapp-s1.pdf]

Article DOI: http://dx.doi.org/10.3201/eid2109.150461

Functional Immune Reconstitution by Therapy for HIV/Mycobacterial Co-infection

Technical Appendix

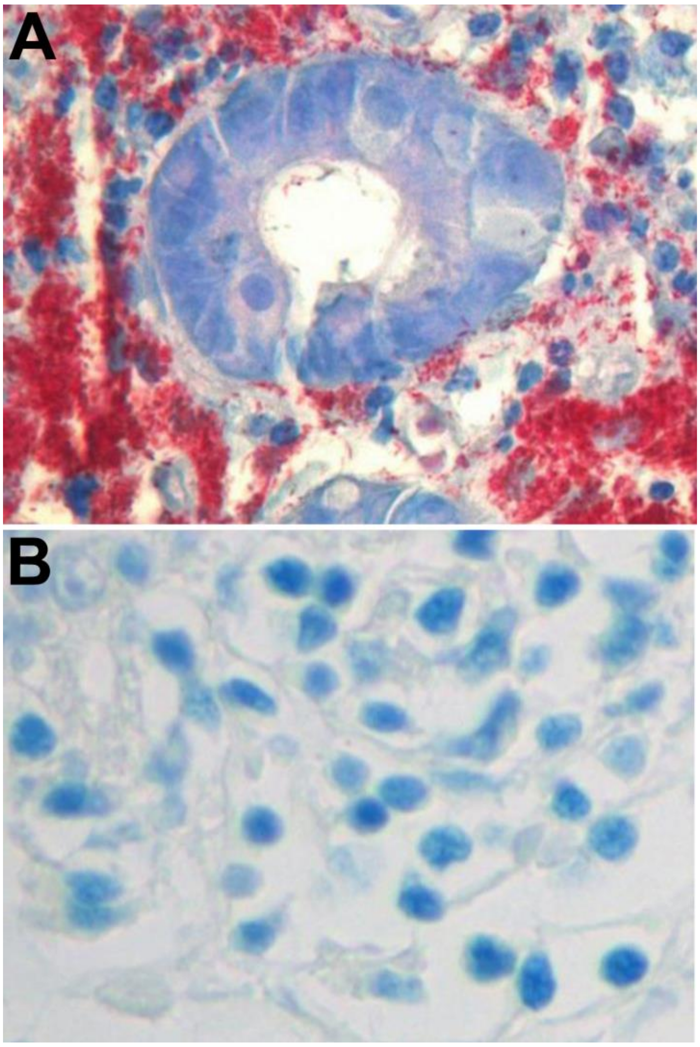

Technical Appendix Figure**.** A) Abundant acid-fast bacilli on a stained histological specimen of a duodenal biopsy before the initiation of adjunctive interleukin-2 (IL-2) immunotherapy in an HIV-infected patient with disseminated *Mycobacterium tilburgii* infection. B) Absence of acid-fast bacilli on a stained histological specimen of a duodenal biopsy after successful adjunctive IL-2 immunotherapy.
